# Supplementary material for: Comparative genomics provides new insights into the diversity, physiology, and sexuality of the only industrially exploited tremellomycete: Phaffia rhodozyma
Source: BMC Genomics. 2016 Nov 9;17:901. doi: 10.1186/s12864-016-3244-7 (PMC5103461; doi:10.1186/s12864-016-3244-7)
Supplement: Additional file 6: — List of orphan genes with links to PFAM (related to Additional file 1: Table S1). (ZIP 1428 kb) [file 12864_2016_3244_MOESM6_ESM.zip › PFAM_UNIQUE/G04394_P.html]

#                                                                            --- full sequence --- -------------- this domain -------------   hmm coord   ali coord   env coord
  
# target name        accession   tlen query name           accession   qlen   E-value  score  bias   #  of  c-Evalue  i-Evalue  score  bias  from    to  from    to  from    to  acc description of target
  
#------------------- ---------- ----- -------------------- ---------- ----- --------- ------ ----- --- --- --------- --------- ------ ----- ----- ----- ----- ----- ----- ----- ---- ---------------------
  
G04394\_P             -            663 AAA\_13               PF13166.1    712       6.1    3.8  10.2   1   1     0.056       8.9    3.3  10.2   119   202   131   207   103   251 0.43 - AAA domain  
G04394\_P             -            663 Asp-B-Hydro\_N        PF05279.6    243       3.5    6.1  10.1   1   1     0.014       6.2    5.2  10.1   118   194   132   204   106   251 0.52 - Aspartyl beta-hydroxylase N-terminal region  
G04394\_P             -            663 BTV\_NS2              PF04514.7    363         8    3.9   9.6   1   1     0.024        13    3.2   9.6   197   268   129   198    95   211 0.53 - Bluetongue virus non-structural protein NS2  
G04394\_P             -            663 Bud13                PF09736.4    145       8.1    5.4   9.2   1   1     0.033        20    4.1   9.2    17    73   134   193   120   235 0.44 - Pre-mRNA-splicing factor of RES complex  
G04394\_P             -            663 CALCOCO1             PF07888.6    546       8.6    3.2   7.3   1   1     0.036        17    2.2   7.3   430   519   132   223   117   244 0.67 - Calcium binding and coiled-coil domain (CALCOCO1) like  
G04394\_P             -            663 DDRGK                PF09756.4    188       1.8    6.6  15.2   1   1     0.008       3.7    5.6  15.2    29    96   133   195   113   205 0.46 - DDRGK domain  
G04394\_P             -            663 DUF2031              PF09592.5    228      0.51    8.1   8.3   1   1   0.00055      0.83    7.4   8.3   107   181   138   211   129   254 0.78 - Protein of unknown function (DUF2031)  
G04394\_P             -            663 GAS                  PF13851.1    201       9.9    4.0   8.6   1   1     0.085        16    3.2   8.6    82   129   132   179   128   183 0.90 - Growth-arrest specific micro-tubule binding  
G04394\_P             -            663 GBP\_C                PF02841.9    297       4.1    5.1   6.6   1   2     0.037        32    2.2   7.9   201   255   132   184   112   208 0.56 - Guanylate-binding protein, C-terminal domain  
G04394\_P             -            663 GBP\_C                PF02841.9    297       4.1    5.1   6.6   2   2       1.1   9.4e+02   -2.6   0.0   153   179   619   646   614   656 0.84 - Guanylate-binding protein, C-terminal domain  
G04394\_P             -            663 Glyco\_hydro\_65N      PF03636.10   255      0.12   10.3   2.5   1   1   3.6e-05      0.22    9.5   2.5   200   253   134   187    73   189 0.84 - Glycosyl hydrolase family 65, N-terminal domain  
G04394\_P             -            663 MAP7                 PF05672.6    171       2.2    6.3  20.8   1   2   0.00013     0.034   12.2  13.3     2    66   114   179   113   201 0.82 - MAP7 (E-MAP-115) family  
G04394\_P             -            663 MAP7                 PF05672.6    171       2.2    6.3  20.8   2   2         9   2.2e+03   -3.5   0.8   140   149   274   283   269   297 0.41 - MAP7 (E-MAP-115) family  
G04394\_P             -            663 Pox\_Ag35             PF03286.9    200       2.4    6.3  17.6   1   2    0.0082       2.9    6.0  14.7    54   118   136   202    79   220 0.60 - Pox virus Ag35 surface protein  
G04394\_P             -            663 Pox\_Ag35             PF03286.9    200       2.4    6.3  17.6   2   2      0.72   2.5e+02   -0.3   0.0   105   137   325   357   254   370 0.46 - Pox virus Ag35 surface protein  
G04394\_P             -            663 REV                  PF00424.13    92     0.045   12.3   4.7   1   2     0.022        67    2.2   1.1    34    71   140   182   121   202 0.52 - REV protein (anti-repression trans-activator protein)  
G04394\_P             -            663 REV                  PF00424.13    92     0.045   12.3   4.7   2   2   4.6e-05      0.14   10.8   0.1    23    46   260   284   243   301 0.79 - REV protein (anti-repression trans-activator protein)  
G04394\_P             -            663 TAP42                PF04177.7    340       3.1    5.3   5.8   1   1    0.0058       5.8    4.4   5.8   124   225   117   217    91   262 0.72 - TAP42-like family
